# Serial measurements of serum PDGF-AA, PDGF-BB, FGF2, and VEGF in multiresistant ovarian cancer patients treated with bevacizumab

**DOI:** 10.1186/1757-2215-5-23

**Published:** 2012-09-19

**Authors:** Christine Vestergaard Madsen, Karina Dahl Steffensen, Dorte Aalund Olsen, Marianne Waldstrøm, Maja Smerdel, Parvin Adimi, Ivan Brandslund, Anders Jakobsen

**Affiliations:** 1 Department of Oncology, Vejle Hospital, Vejle, Denmark; 2 Department of Clinical Biochemistry, Vejle Hospital, Vejle, Denmark; 3 Department of Pathology, Vejle Hospital, Vejle, Denmark; 4 Institute for Regional Health Services Research, University of Southern Denmark, Odense, Denmark; 5 Department of Oncology, Vejle Hospital, Kabbeltoft 25, DK 7100, Vejle, Denmark

## Abstract

**Introduction:**

Anti-VEGF treatment has proven effective in recurrent ovarian cancer. However, the identification of the patients most likely to respond is still pending. It is well known that the angiogenesis is regulated by several other pro-angiogenic proteins, e.g. the platelet - derived growth factor (PDGF) system and the fibroblast growth factor (FGF) system. These other signaling pathways may remain active or become upregulated during anti-VEGF treatment.

The aim of the present study was to investigate if potential changes of PDGF-BB, PDGF-AA, and FGF2 before and during bevacizumab treatment had predictive value for early progression or survival. Furthermore, we wanted to investigate the importance of serum VEGF in the same cohort.

**Methods:**

This study included 106 patients with chemotherapy-resistant epithelial ovarian cancer who were treated with single agent bevacizumab as part of a biomarker protocol. Patients were evaluated for response by the Response Evaluation Criteria In Solid Tumors (RECIST) and/ or Gynecologic Cancer Intergroup (GCIG) CA125 criteria. Serum samples were collected at baseline and prior to each treatment. FGF2, PDGF-BB, PDGF-AA were quantified simultaneously using the Luminex system, and VEGF-A was measured by ELISA. Eighty-eight baseline samples were avaliable for FGF2, PDGF-BB, PDGF-AA analysis, and 93 baseline samples for VEGF.

**Results:**

High baseline serum VEGF was related to poor overall survival. Furthermore, high serum PDGF-BB and FGF2 was of prognostic significance. None of the markers showed predictive value, neither at baseline level nor during the treatment.

## Introduction

The crucial importance of angiogenesis in tumor growth [1,2] has made it a very attractive target for anti-cancer treatment [3]. In particular, focus has been on the vascular endothelial growth factor (VEGF) system due to its central role in the angiogenic process. Over the last few years, bevacizumab, a monoclonal antibody against VEGF, has proven effective in ovarian cancer treatment, both in the frontline setting combined with chemotherapy [4,5] and in recurrent platinum-sensitive [6] and resistant ovarian cancer [7-9]. Recently, data presented at ASCO (American Society of Clinical Oncology) 2012 illustrated a clear improvement in progression-free survival (PFS) for platinum-resistant ovarian cancer patients treated with combined chemotherapy and bevacizumab compared to patients only treated with chemotherapy [10] (the AURELIA study). However, it is well known that only a subgroup of patients will benefit from bevacizumab. Furthermore, the treatment is costly and has some rare but serious side effects. Taken together, there is an obvious need for validated biomarkers [11-13] as a rational basis for patient selection.

A number of studies have demonstrated a relation between high pre-operative baseline serum VEGF and poor prognosis in ovarian cancer in either univariate or multivariate analysis [14-16] and two studies, including the earlier results from our cohort, have demonstrated a similar prognostic relation in patients with recurrent disease prior to commencing bevacizumab treatment [17,18].

One of the major problems during anti-VEGF treatment seems to be tumor evasion from VEGF blockage that may involve several complex escape mecha-nisms [12,13,19,20] including activation of other pro-angiogenic factors such as the fibroblast-growth factor (FGF) and/ or the platelet-derived growth factor (PDGF) system [12,13,19-21]. The PDGF and FGF systems are believed to interact mutually [22] and contribute with different effects to angiogenesis and the tumor microenvironment. FGF2 promotes endothelial cell proliferation and migration [23] whereas PDGF-BB affects pericyte recruitment and stabilization of the vasculature [24-27]. PDGF-AA may also participate in recruitment of the tumor associated stroma that produces angiogenic factors [3,28]. The vasculature in tumors that evade the anti-VEGF treatment may express a higher level of PDGF-BB and develop changes in vessel morphology [29].

The potential clinical importance of FGF or PDGF to tumor growth during bevacizumab treatment remains to be elucidated. It has been suggested that identification of early escape mechanisms could form the basis of changes in the treatment regime [12,13], which makes it interesting to examine factors influencing these mechanisms.

The aim of the present study was to investigate PDGF and FGF serum levels before and during treatment and to investigate whether their potential changes during the treatment had predictive value for early progression. Furthermore, we wanted to investigate the importance of serum VEGF in this larger cohort.

## Materials and methods

### Materials

The study included 106 patients with chemotherapy-resistant epithelial ovarian cancer who were treated with single agent bevacizumab as part of a marker protocol in the period from July 2007 to February 2012. The patients had verified progression of the disease at the time of inclusion in the protocol. At the time of inclusion, paraffin-embedded formalin-fixed tissue and slides from the primary operations were collected and underwent central review by a gyneco-pathologist. Bevacizumab was contraindicated in cases of tumor invasion into the major vessels, whereas bowel involvement was considered a relative contraindication. All patients were evaluable for response by either RECIST [30] and/or GCIG CA125 criteria [31]. CT scan was performed at baseline and at every third cycle, and CA125 was measured prior to each cycle. Patients received bevacizumab 10 mg/kg every third week and continued until progression, side-effects, or patients refusal. The protocol was approved by the Regional Scientific Ethical Committee for Southern Denmark. Written informed consent was obtained from all patients and the Helsinki II Declaration was strictly observed. Blood samples for biomarker analyses were collected serially before each cycle. After half an hour of coagulation, the blood samples were centrifuged at 2000 g for 10 minutes at room temperature and serum was subsequently stored at −80°C until use.

### Methods

#### Luminex

PDGF-AA, PDGF-BB, and FGF2 were quantified simultaneously using the commercial Fluorokine MAP multiplex kits (cat#LAN000 R&D systems, Minneapolis, MN, USA) on the Luminex analyzer (Luminex Corporation, Austin, USA). The serum samples were diluted x 5 in sample diluent provided with the kit. 100 μL of standard, control, and diluted samples were added to the plate together with 50 μL of the antibody capture bead mixture, and the plate was incubated for 2 hours. Then washing was carried out three times using assay buffer and vacuum filtration. Fifty μL of diluted biotin-coupled antibody cocktail were added to each well and the plate was incubated for 1 hour followed by washing. Fifty μL diluted Streptavidin conjugated with phycoerythrin were added to the plate and incubated for 30 minutes in dark. Finally after washing, 100 μL assay buffer were added and the plate was incubated for 2 minutes, after which the analysis was carried out on the Luminex analyzer. All incubations were performed on a plate shaker at room temperature.

PDGF-AA, PDGF-BB, and FGF2 concentrations were determinated from three different standard curves showing MFI (Median Fluorescence Intensity) vs. protein concentrations.

The total coefficients of variation (CV) were determined from one in house serum pool. CV was 6.9% for PDGF-AA, 11.6% for PDGF-BB, and 21.8% for FGF2. In cases where FGF2 was below the detection limit of 16.8 pg/ml (corrected for dilution), ½ * LOD (8.00 pg/m), was chosen and inserted as a value (four samples at baseline, three samples before cycle 2, six samples before cycle 3 and 2 samples before cycle 4).

### ELISA

Serum VEGF was measured by Quantikine Human VEGF Immunoassay (cat#DVE00, SVE00, PDVE00, R&D systems. The performance of the VEGF-ELISA analysis has previously been described in the paper dealing with the first included patients of the protocol (2007–2009) [17]. The total coefficients of variation (CV) were determined from one in house serum pool and three controls of low, medium, and high concentration (RnDSystems), and were found to be between 11%-19%.

### Statistics

The concentrations of the biomarkers did not fit a gaussian-distribution. Correlations between PDGF-AA, PDGF-BB, FGF2, and VEGF were described by Spearman rank correlation coefficient (*r*). Mann Whitney U test was used for comparing the medians between the patients groups (disease control vs. progression) whereas Wilcoxon signed-rank test was used for comparing the medians within the groups. Progression-free survival (PFS) was calculated from start of bevacizumab treatment until progression or death from any cause. The date of progression was reported for both RECIST alone as well as for a combination of RECIST and CA125 (whichever came first). Overall survival (OS) was calculated as the interval from start of bevacizumab treatment until death from any cause. Kaplan-Meier estimates were used for univariate overall survival analysis (OS), illustrated by survival plots, and the log-rank test was used for comparing the survival between two groups. A p-value ≤ 0.05 was considered statistically significant. Number Cruncher Statistical System (NCSS), version 2007, (Kaysville, UT, USA) software package was used for the statistical analyses.

## Results

### Patient characteristics

It appears from Table 1 that serous adenocarcinoma was the most frequent histological subtype (89%). In three patients, the primary ovarian tumors were diagnosed as serous borderline tumors, two with invasive implants (FIGO III ) and one without invasive implants (FIGO I). All three patients had recurrence and progression with invasive implants.

**Table 1:** Patient characteristics

**Number**	**N (%)**
**Total**	106 (100)
**Age at diagnosis, median**	57
**FIGO stage**	
I	8 (8)
II	8 (8)
III	76 (71)
IV	14 (13)
**Histopathological subtype**	
Serous*	94 (89)
Endometrioid	5 (4)
Serous + clear cell (mixed)	1 (1)
Transitiocellular carcinoma	1 (1)
Carcinosarcoma	1 (1)
Adenocarcinoma	1 (1)
Borderline (with invasive implants)	3 (3)
**Number of prior chemotherapy regimens**	
Median	4
Range	2 - 7
**Interval from last chemotherapy regimens**	
Median	1.8
Range	0.3-14.7 months
**Bevacizumab infusions**	
Median	4.5
Mean	7.1
Range	1 – 64**
≤ 2 cycles	22 (21)
≥ 9 cycles	29 (27)
**Performance status at start of bevacizumab treatment**	
0	40 (38)
1	52 (49)
2	13 (12)
3	1 (1)
**Baseline VEGF**, median (range)	504 (27–1847)
**Baseline PDGF-AA**, median (range)	2074.25 (207 – 5476.5)
**Baseline PDGF-BB**, median (range)	7789 (670–28487)
**Baseline FGF2**, median (range)	60 (8–234)

* including primary peritoneal and fallopian cancer.

** still receiving bevacizumab.

Median age at diagnosis was 57 years. At time of refer-ral for bevacizumab treatment, patients had received a median of 4 different chemotherapy regimens (range 2–7), but most of them were still in a good performance stage (PS 0/1 87%). The median number of bevacizumab infusions was 4.5 (range 1–64). At time of analysis (April 2012), 9 patients were still receiving bevacizumab. Twenty-nine (27%) received at least 6 months of treatment (9 cycles), and seven patients (7%) received bevacizumab for at least one year (18 cycles).

### Efficacy

For all included patients, median PFS was 3.9 months (95% C.I. 3.0- 4.2) using the combination of CA125 or RECIST, and 4.2 months (95% C.I. 3.5- 4.8) using the RECIST criteria only. Median OS was 7.7 months (6.3 -8.2). Response rate (RR) was 21% according to the GCIG CA125 criteria. Partial response (PR), evaluated by RECIST, was seen in only 5% of the patients, but 52% of the patients had stable disease (SD) in an intention- to- treat analysis.

### Side effects

During bevacizumab treatment, five patients (4.7%) experienced a gastrointestinal perforation, four patients (3.8%) had a venous tromboembolic event, one patient (0.9%) a cerebral embolism, and another patient (0.9%) an episode of transient ischemic attack (TIA). Four patients (3.8%) suffered from an ileo-vaginal fistula. A urine dipstick test prior to each treatment showed protein 2+ in 25 of the patients (23.6%), but none of the patients presented >; 1 g protein/ 24 h in the following urine collection. Hypertension [32] grade 2, stage 1, (140-159/90-99 mm Hg) was seen in 20 (18.7%) of the patients, in three of them it was present before starting treatment and in 12 of the cases it was only temporary. Hypertension grade 3, stage 2, (160/100 mm Hg) was measured in three of the patients (2.8%) at some point during the treatment.

### Serum PDGF-AA, PDGF-BB, FGF2, and VEGF at baseline

Baseline sera PDGF-AA, PDGF-BB, and FGF2 were available in 88 of the patients and for VEGF in 93 of the patients. Medians and ranges are reported in Table 1 and the correlation coefficients between the different markers are illustrated in Table 2.

**Table 2:** Correlation coefficients between the different markers

	**PDGF-AA**	**PDGF-BB**	**FGF2**
**PDGF-BB**	r = 0.80 (p < 10^-5^)		
**FGF2**	r = 0.42 (p < 10^-5^)	r = 0.32 (p = 0.003)	
**VEGF**	r = 0.50 (p < 10^-5^)	r = 0.53 (p < 10^-5^)	r = 0.25 (p = 0.02)

**Figure 1:**
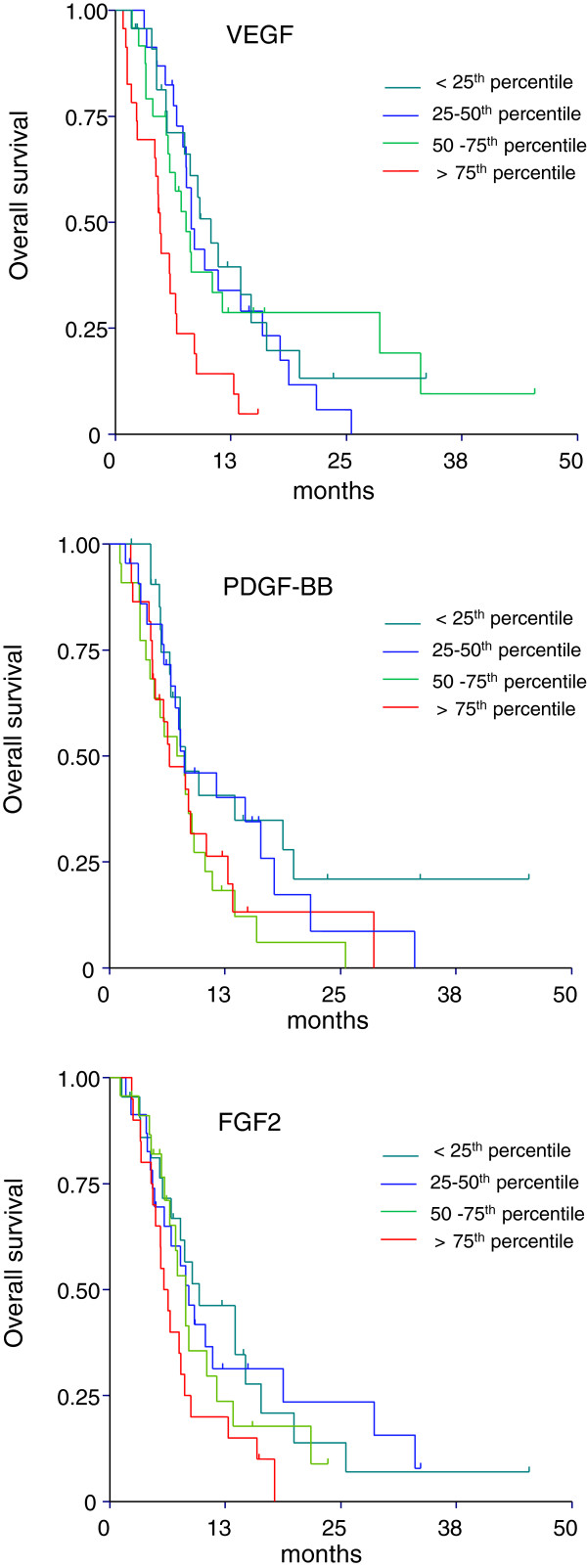
Overall survival curves generated by the Kaplan-Meier method according to quartiles for VEGF, PDGF-BB, and FGF2.

As shown in Figure 1, baseline PDGF-AA, PDGF-BB, FGF2, and VEGF levels were divided into quartiles and investigated in relation to survival.

For VEGF, lower OS was found in patients with levels above the median, p = 0.01 which was further accentuated for patients with levels above the 75^th^ quartile, p = 0.0005.

For PDGF-BB, lower OS was found in patients with levels above the median, p = 0.02, but with no accentuation at the 75^th^ quartile level. The median level of FGF2 had only marginal prognostic significance, p = 0.07, but lower OS was seen in patients with levels above the 75^th^ quartile p = 0.02. PDGF-AA levels did not influence the survival when dividing according to median or the 75^th^ quartile level (data not shown).

The same analyses were made in relation to PFS. Only baseline VEGF above the 75^th^ quartile was related to poor PFS, p = 0.01 (data not shown).

### Serum PDGF-AA, PDGF-BB, FGF2, and VEGF during treatment

**Figure 2:**
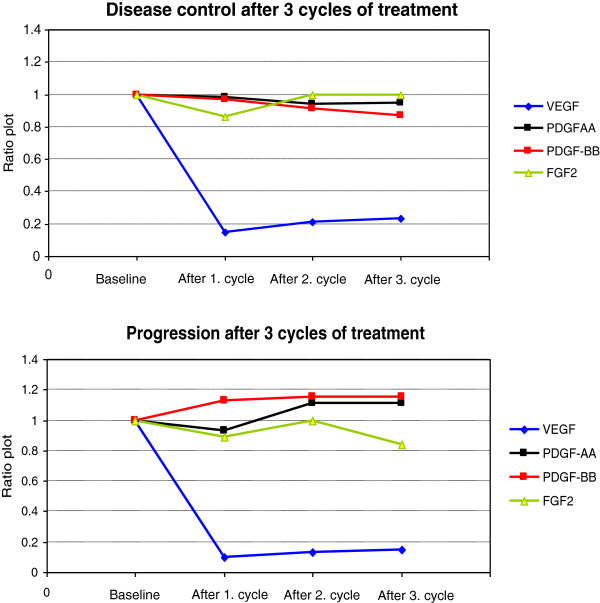
Ratio plot of serum PDGF-AA, PDGF-BB, FGF2, and VEGF from baseline until response evaluation after 3 cycles of bevacizumab.

Serum PDGF-AA, PDGF-BB, FGF2, and VEGF were measured during treatment and investigated in relation to response. Seventy-nine patients were evaluable for response by either RECIST and/or CA125 based on at least 3 serum samples. As illustrated in Figure 2, there were no significant differences of PDGF-AA, PDGF-BB, and FGF2 between the group who progressed (30% of the patients) after three cycles and the group with stable disease (70% of the patients). There were no significant changes in serum medians of PDGF-AA, PDGF-BB, and FGF2 at baseline and before response evaluation within the two groups (data not shown). VEGF decreased from baseline (p < 10^-3^) already after the first cycle of treatment in both groups, and remained unchanged until response evaluation. None of the markers showed significant predictive value.

## Discussion

The present study confirms that bevacizumab is effective as monotherapy in multi-resistant ovarian cancer patients. The adverse event profile is consistent with the findings reported in other comparable studies of bevacizumab in heavily pre-treated ovarian cancer patients [8]. Remarkably, a number of patients (7%), all with measurable disease, were treated for at least one year. There was nothing that separated them from the rest of the patients as regards histopathological characteristics and they had previously received a median of five chemotherapy regimens. One of the patients received the 64th cycle of bevacizumab at the time of the present evaluation. In contrast, a proportion of patients did not benefit at all from the treatment and stopped after short-term treatment due to progression, toxicity or other symptoms.

We found, in accordance with our previous findings [17] and reports from other studies [14-16,18], a relation between high serum VEGF levels and poor OS. Different cut-off values have been used in the literature to dichotomize patients with high and low VEGF values; however, the median VEGF levels are used in most of the cases. However, a study of 312 patients by Hefler et al. [14] clearly demonstrated, on survival plot, an association between preoperative serum VEGF in the upper 4th quartile and poor OS, which is consistent with our results. High levels of PDGF-BB and FGF2 may also be of prognostic importance in heavily pre-treated ovarian cancer patients, and our results are partly in agreement with Schilder et al. [33] who reported a relation between plasma PDGF-AB, PDGF-BB, VEGF and poor OS. However, in the present study we were not able to demonstrate any significant differences of PDGF-AA, PDGF-BB, and FGF2 between patients responding and patients not responding to the treatment.

The decline in serum VEGF during bevacizumab treatment in ovarian cancer patients is in line with the studies by Han et al. [18] and Karihtala et al. [34]. It should be noted that Han et al. [18] also investigated plasma VEGF but without finding any significant changes in plasma VEGF during the treatment. The predictive value of baseline VEGF reported in the first cohort of our patients with ovarian cancer [17] was not confirmed in the present study. This disparity clearly illustrates the drawback of small studies. The risk of such positive results being false positive is very high.

Concentrations of PDGF and FGF in serum or in plasma during anti-cancer treatment have not yet been fully investigated although a decline in serum PDGF-AA or PDGF-BB has been demonstrated in various cancer types during chemotherapy and/or radiotherapy [35-37], and when combined with multi-tyrosine kinase inhibitors (VEGFR-2, FGF and PDGF) [38]. Other reports suggest that plasma PDGF-AB, PDGF-BB, and VEGF may be stable during treatment with Imatinib Mesylate [33]. Associations between dynamics or baseline levels of PDGF and/or FGF and treatment response have been reported in metastatic colorectal cancer (mCRC) [35], breast cancer [39], and in metastatic melanoma [36].

It is obvious that the dynamics of the angiogenic markers are influenced by the specific type of anticancer treatment. To the best of our knowledge, this study is the first to report serum PDGF and FGF measurements during single agent bevacizumab treatment in ovarian cancer. The markers presented here did not show predictive information on early progression and the clinical value of monitoring these markers during bevacizumab therapy seems limited. However, it should be noted that our study used ’single agent targeted treatment’ and that the PDGF system may react differently to cytostatics alone or in combination. Similar reservations apply to the combination of biological treatment and conventional chemotherapy. Furthermore, our patients had received several lines of treatment and marker dynamics may differ between first line and subsequent lines of chemotherapy.

In conclusion, the present study confirms the prognostic importance of baseline serum VEGF and also suggests a prognostic value of serum PFGF-BB and FGF2. The results call for further studies in the early line of treatment.

## Competing interests

The authors declare that there are no conflicts of interest.

## Authors’ contributions

CVM contributed with data collection, data analyses, and manuscript writing. AJ and KDS contributed with study design, data analyses and manuscript writing. MS participated in study design and data collection. DO and IB were responsible for the laboratory analyses. MW performed histological re-examination and participated in drafting the manuscript. PA was involved in the clinical work, coordination and data collection. All authors have read and approved the manuscript.
